# The Role of Social Capital on the Health Promotion Behaviors in Women Teachers at Bam Girls’ Schools

**DOI:** 10.25122/jml-2019-0066

**Published:** 2019

**Authors:** Pourtaheri Asma, Akbarian Bafghi Mohammad Javad, Mohammadi Behzad, Rajabalipour Mohammad Reza

**Affiliations:** School of Public Health, Bam University of Medical Sciences, Bam, Iran

**Keywords:** lifestyle, social capital, teachers, health practice for lifestyle

## Abstract

A healthy lifestyle prevents more than half of diseases and disorders in humans. Social capital is a decisive factor in lifestyle modification. The present study aimed to investigate the effect of social capital on health promotion behaviors in a group of teachers from Bam, Kerman, Iran.

The present study was a descriptive-analytic cross-sectional study. The participants were 245 secondary school teachers that were studied using census data. The integration of two standard questionnaires in the field of health promotion behaviors and social capital were the research instruments. Data analysis was done using the SPSS v23 software.

The average age of the participants was 39.4 years. Also, none of the demographic variables had a significant effect on health promotion behaviors. The highest and lowest score of practices were related to religion believes with an average of 14.47 and physical activities with an average of 10.64. Finally, the average of health promotion behaviors and social capital in women teachers were evaluated at the desirable level.

Considering the role of teachers in educating and providing knowledge to future generations, it can positively affect the health of the whole society through effective interventions on this group. This requires paying more attention to the empowerment and promote the individual and groups of teachers. Therefore, one of the natural and inexpensive ways to improve society’s health is to promote health components in teachers and their self-sufficiency in the field of health.

## Introduction

Despite the practices of the past decades regarding the control of contagious diseases, in recent years we have been struggling to improve lifestyle and change the risky behaviors in order to control chronic diseases; in fact, the current aim of the health system of different countries is to reform people’s lifestyle[1] because lifestyle is one of the critical factors in changing health indicators of a country [2]. Health practice behaviors are a set of actions that are adopted to keep and increase the health of the individual and society [3]. These behaviors are measured in terms of physical activity, stress management, religious activities, interpersonal conversation, responsibility in health, and nutrition [4]. Since behavior change in people is entirely voluntary, health policymakers are looking for approaches to persuade the public and attract people to change their behavior [4,5]. One of the approaches is the social approach and the use of people’s capacity to empower themselves [6]. Of course, to achieve this goal, there is a need for stable and practical resources such as the social capital [7,8]. Today, this concept is considered a vital element besides sociology topics in the health field and has a broad function in sociology [6,7,9]. Communication, trust, networks of friendship, and intimacy of the society members are among the essential components of social capital [10,11]. Recent researches showed that social capital could ensure health practice and facilitate access to a healthful lifestyle [12]. Social capital creates a network of social ties, through which it strengthens interpersonal relations and increases trust between planners and different society classes [12,13]; this trust makes it easier to accept the right norms of life [14]. Social capital also provides policymakers with a reliable source for implementing health policies [15]. Related studies in the field of sociology showed that the level of social capital in Iran is lower than the global average [16, 17]. In developing countries such as Iran, the lack of information and the lack of studies in this field seem to have made it unnecessary to use the potential of social capital in the field of health [17]. Also, according to the critical role of teachers in educating society, if this group is strengthened and empowered, it can have a significant influence on the correct formation of future generations [18]. Therefore, the present study aimed to investigate the role of social capital on the health promotion behaviors in teachers of Bam city.

## Material and Methods

The present study was a cross-sectional study. The participants were 245 female teachers from Bam city.

### Sampling method

Samples were selected by using the census method. The willingness to participate in the study was a criterion for teachers to enter. The exit criterion was also a distorted response.

### Data collection instrument

The data collection instrument was a combination of two standardized questionnaires: Second Edition of Health Promoting Lifestyle Profile II (HPL-PII) and Bullen-Onyx Social Capital Questionnaire (2000). The second version of the health-promoting lifestyle profile is the modified version of HPLP, which was presented by Walker et al. in 1987. The validity and reliability of both questionnaires were demonstrated in similar previous studies. The final questionnaire was presented in three general sections. The first part at the end of the questionnaire contained questions about the demographic variables such as age, family members, education level, husband’s job, and family income. The second part of the questionnaire contained questions about various components of a health-promoting lifestyle, which was evaluated through a Likert scale (always, often, rarely and never). The questionnaire consisted of 52 questions about 6 sub-in structures of nutrition, physical activity, responsibility in health, stress management, interpersonal communication, and religious beliefs. The third part also contained questions related to social capital assessment. The social capital questionnaire had 36 questions that assessed the network structure of social capital in detail and by using the Likert scale (always, often, rarely and never). Questions were placed in the following eight groups of social participation: trust, neighborhood relations, family relationships, living standards, social performance, acceptance of differences, and working relationships.

### Statistical tests and data analysis method

Data were analyzed using the SPSS v23 software, mean and standard deviation, t-test, and chi-square for measuring the meaning of the components and the Pearson correlation coefficient for measuring the relationship between health promotion behaviors. The significance level of all tests in this study was 0.05.

### Ethical considerations

The ethical committee of the Bam University of Medical Sciences approved this study (IR.MUBAM.REC.1397.026). The necessary permissions were obtained to complete the questionnaires in coordination with the Bam Education Department. All aspects of ethical considerations were explained to the participants. In addition, demographic variables and the survey questions were designed completely non-sensitive in order to increase the trust of teachers and ensure that they will remain anonymous.

## Results

Data analysis showed that the replication was 96% among the 245 teachers participating in the study. The average age of the participants was 39.4 years, with a deviation of 7.7 years. Most of the participants had a bachelor’s degree (63.7%), and about 30% had a family member number higher than four. Also, about 82% reported having an average family income. Table 1 shows the data related to the participants’ variables (Table 1).

**Table 1:** Demographic variables of the study participants.

Demographic Variables		Frequency (%)
**Age**	Under 30 years old	26 (10/6%)
	30 to 40 years old	68 (27/8%)
	40 to 50 years old	135 (55/1%)
	50 to 60 years old	16 (6/5%)
**Education level**	Diploma	7 (2/9%)
	Associate diploma	53 (21/6%)
	Bachelor	156 (63/7%)
	Post graduate	29 (11/8%)
**Family members**	Less than three	19 (7/8%)
	Three	54 (22/00%)
	Four	98 (40/00%)
	More than four	74 (30/2%)
**Family Income level**	Less than the mean of other families	35 (14/3%)
	Moderate	192 (82/4%)
	More than the mean of other families	8 (3/3%)
**Wife job**	Employee	91 (37/1%)
	Business person	74 (30/2%)
	Retired	9 (3/7%)
	Others	71 (29/00%)

**Figure 1:**
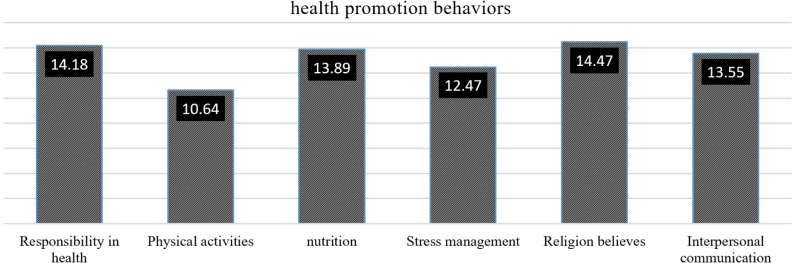
Average scores of different health promotion behaviors.

Comparing the average scores of different structures of health promotion behaviors, it was found that the highest scores were related to religious activities and responsibility and were 14.47 and 14.18, respectively; the lowest score was related to physical activity with mean of 10.64%. Table 2 shows the mean and standard deviation of the scores obtained for the components of health promotion behaviors (Table 2 and Figure 1).

**Table 2:** Mean and standard deviation of health promotion behaviors.

Variable and substructure	Minimum	Maximum	Mean (Sth. Deviation)	p-value
**Responsibility in health**	7.78	20.00	14.18 (2.7)	>0/0001
**Physical activities**	4.38	20.00	10.64 (3.42)	
**Nutrition**	6.11	20.00	13.89 (2.56)	
**Stress management**	5.00	19.44	12.47 (2.96)	
**Religion believes**	6.11	20.00	14.47 (3.26)	
**Interpersonal communication**	5.63	20.00	13.55 (2.85)	

**Figure 2:**
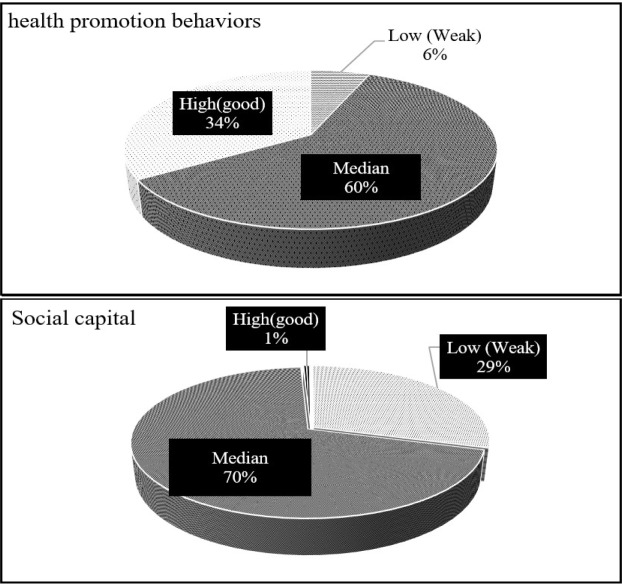
The level of social capital and health promotion behaviors among study participants.

Regarding the average obtained scores, health promotion behaviors were evaluated in approximately 60% of the participants, and the average social capital was evaluated in 70% of them. Eventually, the level of the social capital and health promotion behaviors were desirable in the participants. Table 3 shows the level of two variables with the following behaviors (Table 3 and Figure 2).

**Table 3:** Status of social capital and health promotion behaviors.

Variable and substructure	level	Frequency (%)	p-value
**Responsibility in health**	Low (Weak)	8 (3/30%)	>0/0001
	Median	118 (48/20%)	
	High(good)	119 (48/50%)	
**Physical activities**	Low (Weak)	99 (40/40%)	>0/0001
	Median	103 (42/00%)	
	High(good)	43 (17/50%)	
**Nutrition**	Low (Weak)	8 (3/30%)	>0/0001
	Median	124 (50/60%)	
	High(good)	113 (46/10%)	
**Stress management**	Low (Weak)	40 (16/40%)	>0/0001
	Median	139 (56/70%)	
	High(good)	66 (26/90%)	
**Religion believes**	Low (Weak)	12 (4/90%)	>0/0001
	Median	113 (46/10%)	
	High(good)	120 (49/00%)	
**Interpersonal communication**	Low (Weak)	22 (9/00%)	>0/0001
	Median	114 (46/50%)	
	High(good)	109 (44/50%)	
**Health promotion behaviors**	Low (Weak)	15 (6/10%)	>0/0001
	Median	147 (60/00%)	
	High(good)	83 (33/90%)	
**Social capital**	Low (Weak)	72 (29/40%)	>0/0001
	Median	171(69/80%)	
	High(good)	2(0/80%)	

The correlation test between the social capital and health promotion behaviors showed that social capital has a direct and strong relationship with the sample, and to any extent, social capital increases health promotion behaviors. Table 4 shows the correlation of social capital with health-promoting behaviors (HPB) using the Pearson coefficient.

**Table 4:** Relationship between social capital and health-promoting behaviors.

Variables		Social capital	Health responsibility	Physical activities	Nutrition	Stress management	Interpersonal communication	Spiritual growth	HPB
**Social capital**	**Pearson. Co**	1							
	**p-value**								
**Health responsibility**	**Pearson. Co**	0.27	1						
	**p-value**	.000							
**Physical activities**	**Pearson. Co**	0.18	0.32	1					
	**p-value**	.004	.000						
**Nutrition**	**Pearson. Co**	0.18	0.34	0.21	1				
	**p-value**	.004	.000	.001					
**Stress management**	**Pearson. Co**	0.26	0.26	0.39	0.24	1			
	**p-value**	.000	.000	.000	.000				
**Interpersonal communication**	**Pearson. Co**	0.36	0.34	0.17	0.25	0.36	1		
	**p-value**	.000	.000	.007	.000	.000			
**Spiritual growth**	**Pearson. Co**	0.29	0.28	0.22	0.34	0.34	0.46	1	
	**p-value**	.000	.000	.000	.000	.000	.000		
**HPB**	**Pearson. Co**	0.31	0.53	0.44	0.54	0.56	0.54	0.57	1
	**p-value**	.000	.000	.000	.000	.000	.000	.000	

## Discussion

The analyzed results showed that the social capital has a significant effect on the general health practices of society. Many studies have also reached the same results and confirm this effect [7,11,15]. Therefore, if components such as social capital can be reinforced through social ties, then it can have a great positive impact on health promotion behaviors [19,20].

In fact, besides the great effect of financial resources and equipment on creating equality in health services [21], social capital is also an effective component in the health practice regarding lifestyle, which should be considered by policymakers in this area [9, 22].

This is a general principle that creates the resources in societies with higher social capital, expansion of trust, positive beliefs, proper valuation, shared interests, and pursued goals, which ultimately lead to the health promotion behaviors of that society [23,24]. In these societies, the link between politics and the public is stronger and responsibility is greater for the specified policies [25]. Scheffler and Brown showed in 2008 that high social capital is a guarantee of encouraging positive behaviors, especially in health [26]. Also, Rouxel et al. showed that the social capital has a significant role in improving the oral health of people [27]. The current study also knows strong social capital as an effective source for adequate health practices.

The analyses of available results showed that the status of health promotion behaviors women teachers from Bam is placed optimally, although its average is slightly lower than the average of the country. Similar studies have also reported moderate and adequate levels of health promotion behaviors in the employees [17,28]. The social capital is also moderate in our group. Based on the existing studies, these two factors can play a complementary role in advancing and promoting each other [9]. It is possible to change the components of health promotion behaviors in societies indirectly and with lower costs and guarantee the health of future generations by promoting these groups and considering the educational role of teachers [15]. Recent studies conducted in Italy and Japan showed that those societies that are healthier and livelier have more correlations and effective relationships, and can create a larger social capital [29,30].

In this study, the highest and lowest scores among the components of health promotion behaviors were obtained by religious activities and physical activities, respectively. In a similar study performed in Yazd, the highest score was related to religious activities [31]. Also, religious activities in Isfahan have received a relatively high score among the components of health practices [32]. It seems that these results are due to the effect of common cultural perspectives and attitudes governing traditional-religious communities. Bam is also known as a traditional religious city [33].

Due to some limitations and exclusions, the facilities and physical places suitable for women, such as special parks or gyms, are either unavailable or restricted [34]. In addition, the time limitation for working women is another reason for reducing the level of physical activity in the teachers’ group [35]. Other studies in this field also showed that the level of physical activity in the staff of government departments is lower than that of other people from society [36-38]. In addition, Abdi et al. assessed in a study conducted in Hamedanthe the physical activity of government employees and lower-ranking members [39].

## Conclusion

The role of teachers in educating the community and young generations is undeniable to anyone. Therefore, effective interventions and impact on this group could have a positive effect on the health of the whole community. Also, if teachers become familiar with social concepts and attitudes, and provide their education on this basis, they can be more successful in teaching. This requires more attention for the empowerment and promotion of the individual and group of teachers. Therefore, one of the easy and inexpensive ways to improve the health of the community is to practice the components of health in the classroom of teachers and their self-sufficiency in the field of health.

## Limitations of the study

This study was conducted on the urban female teacher population with the aim of indirectly measuring their individual and family indicators. Therefore, this study could be done in other target groups such as male teachers and students or villagers, and compare the results with each other. Also, the unfamiliarity of some of the concepts of study to the target group may have led to the distortion of some of the answers. However, in order to solve this problem, researchers and interviewers described many of the concepts repeatedly for the target group while completing the questionnaire.

## Acknowledgment

The authors would like to thank the General Department of Education of Bam for its religious support and cooperation in issuing necessary permits. In addition, all officials and teachers of the schools studied are also appreciated for sincere cooperation. Finally, the Associate degree students of Family Health in the second semester of the academic year (2016-2017) of Bam University of Medical Sciences are particularly appreciated for collecting data and their continuous presence in the studied centers.

## Conflict of Interest

The authors confirm that there are no conflicts of interest.
